# Glycerite of Animal Hypophosphites

**Published:** 1878-08

**Authors:** Elwood C. Lester

**Affiliations:** Philadelphia, Pa.


*ART. III.—Glycerite of Animal Hypophosphites.* By ELWOOD C. LESTER, M. D., of Philadelphia, Pa.

Among the therapeutical novelties which have awakened professional interest during the past five years, phosphorous compounds isolated from brain, are entitled to consideration. While there seems to be nothing more remarkable, mysterious, or quackish in isolating phosphorous compounds from brain, than there is in isolating pepsin from the stomach, ptyalin from the saliva, or pancreatine from the pancreas, as is generally known, the solution of isolated phosphorus compounds were received with grave suspicion.

Dr. Percy in his Prize Essay, Phosphorus (Trans. Am. Med. Association, 1872,) suggested that, notwithstanding he was unaware that any one had used the brain hypophosphites as a remedial agent, he would suppose on hypothetical grounds, that they would prove a valuable preparation of phosphorus because they represented phosphorous in the exact form in which it sustained vital functions." In "New Remedies," November, 1876, appeared the first literature on the subject, and since then these compounds have grown into professional favor. It is estimated that the use of them now exceeds five hundred pounds a month.

Before Churchill of Paris declared that the phosphatic nutrient of the human organism is in the formula of a hypophosphite, chemists generally held the view that phosphorous existed in the human organism only as a phosphate. Churchill, however, found followers.

Dr. Percy, in his Prize Essay, demonstrated that "the phosphatic nutrient of the brain is in the form of an alkaloidal hypophosphite." Francis Gerry Fairfield says that the phosphorus in the human organism is in the form of the phosphates of lime and magnesia; phosphites of ammonia, potash and magnesia; nitrogeneous and oleo-nitrogeneous hypophosphites of lime, potash, soda, ammonia and iron; hypophosphite in combination with albumen, and hydrated hypophosphorous acid in combination with oil. I believe there is no essential difference of opinion as to the phosphorous elements of the human organism between Drs. who have recently written on this subject. Their seeming differences are simply questions of priority in which the profession has no interest.

At a glance it will be seen that these hypophosphites, isolated without heat or any oxidizing agent, will be in the exact formula in which they subserve vital functions. The inference is natural that such hypophosphites must be more exactly adapted to the requirements of the organism in all cases in which there is a deficiency of the phosphorous elements, and are entitled to preference over the laboratory kind whenever hypophosphites are indicated.

The range of pathological conditions in which these are indicated is extensive and constitutes a very serious class of maladies. Recently my experience with the ordinary hypophosphite of calcium has been very satisfactory. A month ago I began testing the Glycinite of the Animal Hypophosphites, and so far the result has been favorable. The period has been too short to decide with any certainty its positive value, but the evidence from many others seems to give assurance that it is equal in Phthisis to any other remedy.

Professor Wellford, of Richmond, Va., in a paper recently read before the Richmond Academy of Medicine, showed "that the trifacial neuralgia of pregnant and nursing women depended upon a deficiency of the phosphorous elements. He had seen teeth of pregnant and nursing women drawn by dentists, which seemed normal in their external appearance, but upon examination after withdrawal consisted of nothing more than enamel. He attributed cases of dental decay, neuralgia, stomatitis materna, and pain in the back occurring in pregnant and nursing women to the demand for phosphates which nature was unable to supply." Others attributed a large per cent of the diseases of dentition to a deficient supply of the phosphates.

Routh, of London, has shown that premature decay of the brain functions, whether resulting from overwork or sexual excesses, is attended by a marked deficiency of protagon. Dr. J. S. Jewell has given very cogent reasons for believing that the great cause of neuralgia is in innutrition of the nerve cells, or, as Romberg has expressed it, the cry of the hungry nerve for blood. Dr. Edward C. Mann has shown that chronic insanity is largely in consequence of brain innutrition. Hentier has demonstrated that the integrity of the brain depended upon a proper supply of the phosphorous principles. But the value of protagon is so well recognized that it scarcely need be debated. It, as Fairfield has said, "is the organizer and tissue generator of the animal and vegetable kingdom, and the source of all the varied morphological phenomena illustrated in the growth of the living bodies from a simple albuminoid base. It is protagon, which co-ordinates its organization into tissues, having special functions, and determines all its various morphological products."

Life has a chemistry of its own. In its alembic are developed compounds that can be only imperfectly imitated in the laboratory. To them is imparted a vital adaptability which the skill of the chemist cannot impart. He can, however, isolate and preserve many of these productions and employ them successfully in cases in which the vital elaboration is insufficient. Pepsin, pancreatin and isolated phosphorous compounds are familiar examples. They fill indications in the management of various forms of innutrition, nothing else can so well reach. They give the products of the vital laboratory, back to the organs and functions, and thus become therapeutical agents of high value. Scarcely less valuable than these is the phosphorous compounds isolated from the vegetable kingdom. These the writer has used largely in his practice during the past year in wasting diseases of children, and the more he has employed them the more is he impressed with their great value.

The Glycerite of Animal Hypophosphites is designed to be the *par excellent* preparation isolated from organic sources.

The following is the composition of one hundred parts as given in the "Philadelphia Druggist and Chemist:"

| | Parts. |
|---|---|
| Isolated Nitrogeneous Hypophosphate of Calcium, | 6 |
| " " " Sodium, | 5 |
| " " " Potassium, | 3 |
| " " " Ammonium, | 8 |
| " " " Magnesium, | 3 |
| " " " Iron, | 4 |
| Hypophosphorous Acid with Albumen, | 2 |
| Zoaline (an alkaloidal hypophosphite composed of three parts of blended nitrogen and glycerine with one of hypophosphorous acid.), | 9 |
| Glycero Hypophosphorous Acid, | 5 |
| Hypophosphorous Acid (liberated from its oleo nitrogeneous association, | 5 |
| Bower's Chemically Pure Glycerine, | 50 |

The dose is ten drops thrice daily.

It is designed to represent all the phosphorous compounds of the animal organism, as the probability is that it contains all that is essential for therapeutical purposes. If tested by the profession I have no doubt but that it will grow into favor and become extensively used. It will prove an excellent nutritive tonic and will meet all the indications that it is possible to meet, with any of the phosphorous compounds.

———:o:———

Deaths from Chloroform.—A case of this occurring in the dentist's chair is reported in the *British Medical Journal* for May 18, 1878; another case will be found in the number for May 25th, and two more, which occurred at the Edinburgh Royal Infirmary, in the issue of June 1st.

